# Comparative Analysis of qSOFA, PRIEST, PAINT, and ISARIC4C Scores in Predicting Severe COVID-19 Outcomes Among Patients Aged over 75 Years

**DOI:** 10.3390/diseases12120304

**Published:** 2024-11-28

**Authors:** Daniela Rosca, Vamsi Krishna, Chandramouli Chetarajupalli, Adelina Maria Jianu, Ilona Emoke Deak, Claudia Raluca Balasa Virzob, Sorina Maria Denisa Laitin, Madalina Boruga, Rodica Lighezan

**Affiliations:** 1 Doctoral School, “Victor Babes” University of Medicine and Pharmacy Timisoara, Eftimie Murgu Square 2, 300041 Timisoara, Romania; daniela.rosca@umft.ro; 2 Sri Devaraj Urs Medical College, Kolar 563101, India; vamsikr2345@gmail.com; 3 College of Medicine, Zhengzhou University, Zhengzhou 450052, China; cmouli24@gmail.com; 4 Department of Anatomy and Embryology, “Victor Babes” University of Medicine and Pharmacy Timisoara, Eftimie Murgu Square 2, 300041 Timisoara, Romania; adelina.jianu@umft.ro; 5 Department of Clinical Nursing, “Victor Babes” University of Medicine and Pharmacy Timisoara, Eftimie Murgu Square 2, 300041 Timisoara, Romania; sukosd.emoke@umft.ro (I.E.D.); virzob.claudia@umft.ro (C.R.B.V.); 6 Discipline of Epidemiology, Department of Infectious Diseases, “Victor Babes” University of Medicine and Pharmacy Timisoara, Eftimie Murgu Square 2, 300041 Timisoara, Romania; laitin.sorina@umft.ro; 7 Department of Toxicology, Drug Industry, Management and Legislation, Faculty of Pharmacology, “Victor Babes” University of Medicine and Pharmacy Timisoara, Eftimie Murgu Square 2, 300041 Timisoara, Romania; 8 Discipline of Parasitology, Department of Infectious Diseases, “Victor Babes” University of Medicine and Pharmacy Timisoara, Eftimie Murgu Square 2, 300041 Timisoara, Romania; lighezan.rodica@umft.ro

**Keywords:** COVID-19, elderly patients, qSOFA, PRIEST score, PAINT score, ISARIC4C score, mortality predictors

## Abstract

Background: Elderly patients, particularly those over 75 years old, have been disproportionately affected by COVID-19, exhibiting higher rates of severe outcomes, such as ICU admissions and mortality. This study aimed to evaluate and compare the effectiveness of various clinical scoring systems—qSOFA, PRIEST, PAINT, and ISARIC4C—in predicting ICU admission, the need for mechanical ventilation, and mortality among elderly COVID-19 patients. Methods: In this retrospective cohort study conducted at two tertiary care hospitals, 131 elderly patients (aged ≥ 75) and 226 younger controls (aged < 65) with confirmed COVID-19 were included. Clinical scores were computed at admission and five days after symptom onset. Kaplan–Meier survival analysis and Receiver Operating Characteristic (ROC) curve analysis were performed to assess the predictive performance of the scores regarding severe outcomes. Results: Kaplan–Meier analysis indicated significantly lower survival probabilities for elderly patients with high scores at admission. Those with an ISARIC4C score above 11.8 had a survival probability of 25% compared to 74% for those below this threshold (*p* < 0.001). Similarly, elderly patients with a qSOFA score above 2.1 had a survival probability of 36% compared to 72% for those with lower scores (*p* < 0.001). The PRIEST and PAINT scores also demonstrated predictive validity; patients with a PRIEST score above 6.3 and a PAINT score above 6.5 at admission showed comparable decreases in survival probabilities. ROC analysis at five days post-symptom onset revealed that the ISARIC4C score had the highest area under the curve (AUC) of 0.772, suggesting excellent predictive validity for severe outcomes, including mortality. The optimal cutoffs identified were 11.2 for ISARIC4C, 6.3 for PRIEST, and 6.5 for PAINT, each displaying high sensitivity and specificity. Conclusions: The ISARIC4C, qSOFA, PRIEST, and PAINT scores are robust predictors of severe outcomes in elderly COVID-19 patients over 75 years old, as confirmed by Kaplan–Meier and ROC analyses. These tools can be crucial for early identification of patients at high risk of adverse outcomes, guiding clinical decision making, and optimizing resource allocation. The use of these scoring systems should be encouraged in clinical settings to enhance the management of elderly COVID-19 patients. Further research is necessary to validate these findings across different populations and settings.

## 1. Introduction

The COVID-19 pandemic has exerted a profound impact on global health systems, with elderly populations bearing a disproportionate burden of morbidity and mortality. Patients aged over 75 years represent a particularly vulnerable group, exhibiting higher rates of hospitalization, severe disease progression, and mortality compared to younger cohorts [1,2,3]. Several factors contribute to this increased susceptibility, including age-related physiological changes, the presence of multiple comorbidities, and diminished immune responses [4,5,6].

Early identification of patients at high risk for severe COVID-19 outcomes is critical for optimizing clinical management and resource allocation. Accurate risk stratification allows for timely interventions, appropriate use of intensive care resources, and potentially improved patient prognoses [7,8]. Clinical scoring systems have been widely used in various medical conditions to predict disease severity and mortality, aiding clinicians in decision-making processes [9,10].

Among the scoring systems applied in the context of COVID-19, the quick Sequential Organ Failure Assessment (qSOFA), the PRIEST COVID-19 clinical severity score, the PAINT score, and the International Severe Acute Respiratory and Emerging Infection Consortium 4C Mortality Score (ISARIC4C) have garnered attention for their potential utility in predicting disease progression and outcomes [11,12,13,14]. These scores incorporate a range of clinical parameters, including vital signs, laboratory results, and patient demographics, to estimate the risk of adverse outcomes.

Previous studies have evaluated the utility of these scoring systems in general COVID-19 patient populations, yielding varying degrees of predictive accuracy [15,16,17]. However, there is a paucity of data specifically assessing the performance of these scores in the elderly. Given the unique clinical presentations and higher risk profiles of this age group, it is imperative to validate and potentially adapt these tools for better risk stratification [18,19].

Assessing the comparative effectiveness of qSOFA, PRIEST, PAINT, and ISARIC4C scores in predicting severe outcomes in elderly COVID-19 patients can inform clinical decision making and improve patient care. Understanding which scores offer the most accurate predictions can help clinicians prioritize patients who may benefit from more aggressive interventions or closer monitoring. Therefore, this study’s objective is to evaluate and compare the predictive utility of qSOFA, PRIEST, PAINT, and ISARIC4C scores at admission and five days after symptom onset in elderly patients aged over 75 years diagnosed with COVID-19, aiming to establish the most accurate score for predicting a severe form of COVID-19. By including a control group of patients under 65 years old, we also aim to contextualize these findings within a younger population. Ultimately, this research seeks to enhance clinical risk stratification and optimize management strategies for elderly patients affected by COVID-19.

## 2. Materials and Methods

### 2.1. Legal and Ethical Considerations

This retrospective cohort study was conducted at the Victor Babes Hospital of Infectious Diseases Timisoara, affiliated with the Victor Babes University of Medicine and Pharmacy, from January 2022 to June 2024. This study was approved by the Institutional Review Board, and written informed consent was obtained from all participants or their legal guardians. This study complied with the ethical standards of the Declaration of Helsinki and relevant regulatory guidelines. All patient data were anonymized to ensure confidentiality. The study protocol was approved by the Institutional Review Board, and written informed consent was obtained from all participants or their legal guardians. This study adhered to the ethical standards of the Declaration of Helsinki, EU Good Clinical Practice Directives (2005/28/EC), and the International Council for Harmonization of Technical Requirements for Pharmaceuticals for Human Use (ICH) guidelines. All patient data were anonymized to ensure confidentiality.

### 2.2. Inclusion and Exclusion Criteria

Inclusion criteria for the elderly group were patients aged 75 years and older with laboratory-confirmed COVID-19 via RT-PCR testing. The control group included patients under 65 years old with confirmed COVID-19. All participants required the availability of data necessary for calculating the specified clinical scores at both time points (admission and five days post-symptom onset). All patients were monitored for a total duration of 21 days (3 weeks). To ensure completeness of the dataset, we included measurements taken at admission and at 5 days. Exclusion criteria included patients aged 65–74 years, incomplete medical records lacking essential data for score calculation, patients who did not consent to participate, and those transferred to or from other facilities, which would hinder reliable follow-up data collection.

### 2.3. Study Variables

Data were collected from electronic medical records and included demographic information (age, gender), comorbidities, COVID-19 vaccination status (those with a minimum of two vaccine shots), smoking and alcohol use, and baseline clinical parameters. The Charlson Comorbidity Index (CCI) was calculated for each patient to assess comorbidity burden. Clinical data necessary for calculating the qSOFA, PRIEST, PAINT, and ISARIC4C scores were collected at admission and five days after symptom onset. Outcomes assessed included severity of COVID-19 (classified as mild, moderate, or severe/critical based on WHO criteria [20]), need for supplemental oxygen, intensive care unit (ICU) admission, mechanical ventilation, length of hospital stay, mortality, and survival time.

Day 5 post-symptom onset was chosen as a practical and clinically relevant time point for assessing scoring systems in elderly COVID-19 patients, even though the trajectory of clinical parameters was uncertain. This choice allowed for capturing the early progression of the disease when outcomes were not yet definitive but were beginning to diverge between patients with severe and less severe courses. The rationale was to evaluate the predictive power of the scoring systems during a dynamic phase of the illness, providing insights into early risk stratification. While later data could offer information on recovery or stabilization, focusing on this earlier period aimed to inform timely clinical decision making, acknowledging the inherent uncertainties in disease progression.

### 2.4. Definitions

The qSOFA score is a simplified tool used to rapidly identify patients at risk of poor outcomes due to infection [21]. It assesses three criteria: (1) altered mentation, a Glasgow Coma Scale score of less than 15; (2) systolic blood pressure ≤ 100 mmHg; (3) and respiratory rate ≥ 22 breaths per minute. (4) Each criterion scores one point, with a total score ranging from 0 to 3. A qSOFA score of 2 or more suggests a high risk of adverse outcomes, such as increased mortality and prolonged ICU stay.

The Pandemic Respiratory Infection Emergency System Triage (PRIEST) score was developed to predict the risk of adverse outcomes in patients with suspected COVID-19 [22]. It includes parameters such as age, sex, respiratory rate, oxygen saturation, heart rate, systolic blood pressure, temperature, and comorbidities. The score ranges from 0 to 34, with higher scores indicating a greater risk of severe disease. The PRIEST score aids in triaging patients and determining the need for hospital admission or higher levels of care.

The Pneumonia Assessment of Infection Severity (PAINT) score is designed to assess the severity of pneumonia, including COVID-19-related pneumonia [23]. It incorporates the following clinical and laboratory findings: age, respiratory rate, systolic blood pressure, oxygen saturation, confusion, urea levels, and C-reactive protein (CRP). The score ranges from 0 to 20, with higher scores indicating more severe disease and a higher risk of mortality. The PAINT score helps in guiding treatment decisions and predicting patient outcomes.

The International Severe Acute Respiratory and Emerging Infection Consortium Coronavirus Clinical Characterization Consortium (ISARIC4C) Mortality Score predicts in-hospital mortality among patients with COVID-19 [24]. It uses eight variables: age, sex, number of comorbidities, respiratory rate, oxygen saturation, Glasgow Coma Scale, urea level, and C-reactive protein (CRP). The score ranges from 0 to 21, with higher scores indicating a higher risk of death. The ISARIC4C score stratifies patients into low, intermediate, high, and very high risk of mortality.

### 2.5. Statistical Analysis

Statistical analysis was performed using SPSS Statistics version 26.0. Continuous variables were presented as means ± Standard Deviation (SD) and categorical variables as frequencies and percentages. Comparisons between the elderly and control groups were conducted using Student’s *t*-test for normally distributed continuous variables and the Mann–Whitney U test for non-normally distributed continuous variables. For categorical variables, Fisher’s exact test was utilized to obtain exact *p*-values. Receiver Operating Characteristic (ROC) curves were constructed to evaluate the predictive accuracy of the clinical scores. The area under the curve (AUC), sensitivity, specificity, and optimal cutoff values were determined using the Youden Index. Kaplan–Meier survival analysis was performed to compare survival times between groups, and the log-rank test was employed to assess statistical significance. Additionally, Cox proportional hazards regression analysis was used to identify independent predictors of mortality. A *p*-value of less than 0.05 was considered statistically significant.

## 3. Results

### 3.1. Patient Demographics

It was found that the elderly patients had a significantly higher average age (78.45 ± 3.12 years) compared to the younger group (52.37 ± 9.54 years, *p* < 0.001). A higher proportion of the elderly were female (60.31% vs. 48.23%, *p* = 0.028). In clinical terms, significant disparities were evident; the elderly had a lower BMI and were more likely to be non-smokers but less likely to use alcohol. Despite similar COVID-19 vaccination rates, elderly patients had more severe outcomes, including higher ICU admissions and mortality rates (ICU admission: 24.43% vs. 10.18%, *p* < 0.001; mortality: 20.61% vs. 4.42%, *p* < 0.001), as presented in Table 1.

### 3.2. Physiological Parameters and Clinical Scores

The elderly presented with lower oxygen saturation (91.23% ± 3.57 vs. 94.58% ± 2.43, *p* < 0.001) and higher respiratory rates (22.84 ± 3.98 breaths/min vs. 19.37 ± 3.27 breaths/min, *p* < 0.001). Clinical severity was also greater in the elderly, as evidenced by higher scores on the Glasgow Coma Scale and critical biomarkers, such as BUN and creatinine levels. Elevated clinical scores including qSOFA, PRIEST, PAINT, and ISARIC4C among the elderly underscored their higher risk at hospital admission (Table 2).

By day five post-symptom onset, the results revealed a continued deterioration in the physiological and clinical scores of elderly patients. Oxygen saturation further declined to 89.26% ± 4.41 and respiratory rates increased to 24.18 ± 4.80 breaths/min (both *p* < 0.001 compared to younger patients). Clinical scores such as qSOFA, PRIEST, PAINT, and ISARIC4C showed marked increases at 5 days post-symptom onset, reflecting the escalating severity of the condition in the elderly cohort (Table 3).

The stratified analysis in Table 4 demonstrates a clear protective effect of COVID-19 vaccination across both elderly (>75 years) and younger (<65 years) populations. Among elderly patients, those who were vaccinated showed significantly lower rates of ICU admission (14.3% vs. 31.1%, *p* = 0.002), oxygen supplementation (38.6% vs. 59.0%, *p* = 0.015), mechanical ventilation (9.8% vs. 21.3%, *p* = 0.010), and mortality (13.2% vs. 25.4%, *p* = 0.003) compared to their unvaccinated counterparts. Similarly, in the control group under 65 years, vaccinated individuals experienced reduced severe outcomes, including ICU admissions (6.2% vs. 12.1%, *p* = 0.002), oxygen supplementation (28.6% vs. 43.4%, *p* = 0.015), mechanical ventilation (4.2% vs. 10.2%, *p* = 0.010), and mortality rates (3.3% vs. 8.7%, *p* = 0.003). Additionally, vaccinated patients in both age groups exhibited significantly lower mean clinical scores across all assessed tools, qSOFA (2.1 ± 0.7 vs. 2.6 ± 0.8, *p* = 0.043), PRIEST (6.1 ± 1.8 vs. 7.5 ± 2.1, *p* = 0.021), PAINT (6.3 ± 2.0 vs. 8.3 ± 2.2, *p* = 0.019), and ISARIC4C (11.6 ± 3.1 vs. 13.4 ± 3.3, *p* = 0.026), in the elderly, and there were similar reductions in the younger control group.

### 3.3. Determination of Optimal Cutoff Values of Negative Outcomes

**Figure 1:**
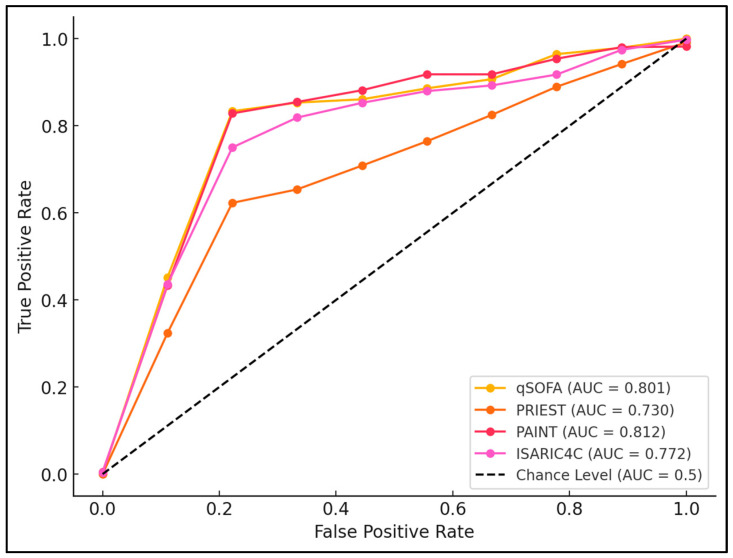
ROC curve analysis.

Notably, a qSOFA score above 2.1 at admission had high sensitivity (82.14%) and specificity (78.95%), with an area under the curve (AUC) of 0.801, indicating strong predictive capability (*p* < 0.001). Similarly, the PAINT score demonstrated excellent predictive performance with a cutoff value of 6.5, achieving a sensitivity of 83.33% and a specificity of 79.31%. Over time, by day five, these cutoff values were adjusted, with qSOFA increasing to 2.5 and PAINT to 7.7, reflecting changing clinical conditions and maintaining significant predictive accuracy (Table 5 and Figure 1).

Regression analysis in Table 6 quantified the risk of developing severe COVID-19 among elderly patients based on exceeding specific clinical score thresholds. At admission, exceeding a qSOFA score of 2.1 was associated with a hazard ratio (HR) of 2.52 (95% CI: 1.73–3.67, *p* < 0.001), indicating more than double the risk of severe outcomes. The ISARIC4C score at admission had the highest HR of 3.02 (95% CI: 2.07–4.41, *p* < 0.001). These associations were similarly strong or increased at five days post-symptom onset, with the NEWS2 score above 11.2 showing an HR of 3.28 (95% CI: 2.19–4.92, *p* < 0.001), highlighting the worsening clinical trajectory for those with elevated scores.

**Figure 2:**
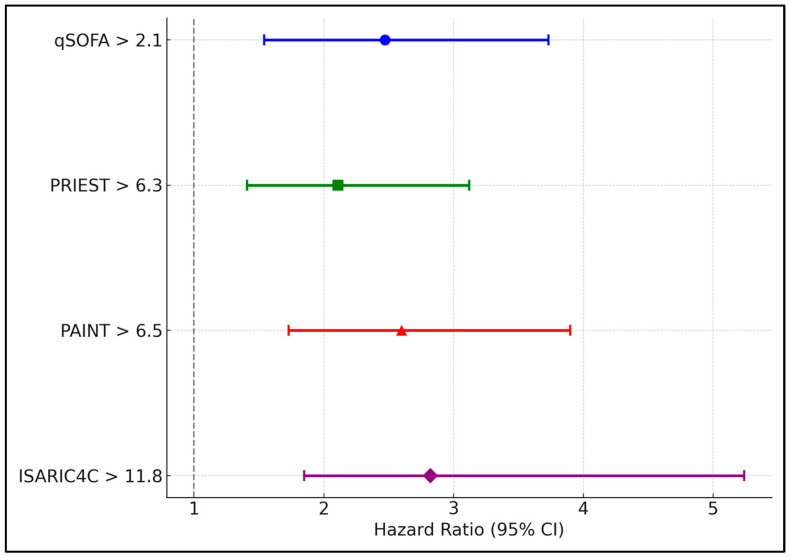
Forest plot analysis of hazard ratio scores in elderly patients.

The findings showed that a qSOFA score greater than 2.1 at admission was associated with an HR of 2.47 (95% CI: 1.54–3.73, *p* < 0.001), indicating a significant increase in mortality risk. This risk escalated across different scores by day five, with the SOFA score above 7.7 associated with the highest HR of 3.34 (95% CI: 2.00–4.50, *p* < 0.001). These results underscore the critical importance of these clinical scores in identifying elderly patients at higher risk of death from COVID-19, supporting the need for timely and aggressive intervention (Table 7 and Figure 2).

## 4. Discussion

### 4.1. Analysis of Findings

The findings from this study underscore the critical role of clinical scoring systems like ISARIC4C, qSOFA, PRIEST, and PAINT in the management and risk stratification of elderly COVID-19 patients. These scores serve not only as indicators of current patient status but also as prognostic tools that can predict the trajectory of COVID-19 complications. The ISARIC4C score emerged as a notably robust predictor, exhibiting the highest sensitivity and specificity for severe outcomes, including mortality. This could be attributed to the comprehensive factors included in the ISARIC4C scoring, which encompass a wider range of clinical symptoms and biomarkers compared to the more focused parameters of qSOFA and PRIEST. The broad spectrum of clinical variables considered by ISARIC4C may capture the complex pathology of COVID-19 more effectively, particularly in a population that is already vulnerable due to age and comorbidities.

Moreover, the utility of qSOFA and PRIEST in this setting prompts a discussion about their applicability across different clinical environments and populations. qSOFA, originally developed for quick sepsis evaluation, has shown its adaptability in gauging severity in other systemic infections, such as COVID-19, highlighting its potential as a universal triage tool in emergency settings. However, its performance, while significant, was not as pronounced as ISARIC4C, suggesting that while useful, qSOFA may require adjunctive scoring systems to improve accuracy in specific patient subsets [25,26]. Similarly, the PRIEST score, which is tailored more towards a general emergency presentation, reaffirms the need for specialized scores in managing elderly populations where typical presentations of diseases may be absent or altered.

The role of the PAINT score in this analysis also sheds light on the evolving landscape of clinical scoring systems. As newer scores like PAINT are developed and validated, there is a potential for them to be refined and perhaps even combined with traditional scores to enhance predictive accuracy and clinical utility. Integrating machine learning algorithms with these scoring systems could potentially offer real-time predictive analytics, improving outcome predictions and enabling personalized patient management strategies. Moving forward, it will be essential to validate the effectiveness of these scores in other cohorts and to assess their performance in prospective studies to ensure they can reliably inform clinical decisions and improve patient outcomes across diverse healthcare settings.

In a similar manner, the study conducted by Heydari et al. [27] involved 894 patients presenting to the emergency department, where the AUROC for qSOFA was 0.799, demonstrating good predictive value for COVID-19 mortality comparable to CURB-65 (0.829) and PSI (0.830) but superior to SIRS (0.759). The demographic data indicated that non-surviving patients were significantly older and had more comorbidities than surviving ones. In a similar manner, the study by Liu et al. [21], which included 140 critically ill COVID-19 patients, found that SOFA, with an AUROC of 0.890, outperformed qSOFA, which had an AUROC of 0.742. This suggests a higher predictive accuracy for SOFA in a critical care setting. While both qSOFA and SOFA are validated tools for predicting mortality, their performance varies significantly with the severity of the patient’s condition upon admission, underlining the importance of choosing an appropriate assessment tool based on patient acuity.

Similarly, Julio Alencar and colleagues investigated the efficacy of SIRS, qSOFA, and NEWS scores for predicting mortality, bacterial infection, and ICU admission among 2473 COVID-19 patients at a major Brazilian hospital [28]. Their findings showed poor performance of the SIRS and qSOFA scores but high sensitivity of the NEWS score in predicting in-hospital death (0.851), early bacterial infection (0.851), and ICU admission (0.868), despite its low specificity. In a similar manner, a study by Cosmin Citu et al. [29] evaluated the predictive value of SOFA and qSOFA scores for in-hospital mortality among 133 patients in Romania, revealing both scores as excellent predictors of mortality with ROC-AUC values of 0.800 for SOFA and 0.794 for qSOFA. Notably, regression analysis indicated that every one-point increase in the SOFA score increased the mortality risk by 1.82 and in the qSOFA score by 5.23.

Another study conducted an extensive assessment of seven risk stratification tools, including NEWS2, PRIEST, and PMEWS, in the Western Cape, South Africa [30]. Among 446,084 patients, 3.45% experienced severe outcomes such as intubation, non-invasive ventilation, death, or ICU admission within 30 days. The tools showed moderate sensitivity and specificity, with a notable clinical decision-making accuracy for inpatient admission marked by a sensitivity of 0.77 and a specificity of 0.88. Despite good estimated discrimination (C-statistic between 0.79 and 0.82), the practical application at recommended thresholds would have significantly increased admissions with minimal reduction in false negatives. In a similar manner, the study by Suh et al. [31] evaluated the modified PRIEST score (mPRIEST) in two New York City EDs, demonstrating that this tool effectively identified patients at very low risk of adverse outcomes, such as death or mechanical ventilation, with a sensitivity of 97.7% for those above the threshold value. The mPRIEST study, though smaller with outcomes available for only 306 patients, suggested strong potential for bedside application in identifying low-risk patients, contrasting with Marincowitz’s findings where the practical utility in reducing admissions was limited.

Regarding the ISARIC4C score, the studies by Joshua M. Riley [32] and Rehab Abd Elfattah Mohamed et al. [33] critically evaluate the performance of the 4C Mortality Score in predicting COVID-19 mortality across different populations and settings. Riley’s study involved 426 patients in an urban United States setting, demonstrating the utility of the 4C Mortality Score with an area under the receiver operator characteristic curve (AUROC) of 0.85, indicating robust predictive capability in a diverse inpatient population (54.5% Black or African American). The study noted a mortality rate of 16.7% among the cohort, underscoring the score’s relevance in pre-vaccine era clinical decision making. In a similar manner, the study by Mohamed et al. in Saudi Arabia assessed the 4C Score’s accuracy among a mixed group of 506 home-isolated and hospitalized COVID-19 patients. Their findings revealed a model with 71% sensitivity and 88.6% specificity, with an AUROC of 0.9. This study also identified hypoxia, high respiratory rate, C-reactive protein, and blood urea nitrogen as significant predictors of mortality within the model, suggesting that these factors are crucial in mortality risk stratification.

The study findings highlight that comorbidities significantly contribute to the poorer outcomes observed in the elderly population compared to younger patients following COVID-19. The most impactful contributors include higher rates of chronic health conditions, as evidenced by the CCI scores, with 65.65% of elderly patients having a CCI > 2 compared to 31.42% in younger patients (*p* < 0.001). Renal dysfunction, reflected in elevated blo BUN and creatinine levels, as well as impaired respiratory function indicated by lower oxygen saturation and PaO_2_/FiO_2_ ratios, were more pronounced in the elderly group. These physiological differences align with elevated clinical scores, such as qSOFA, PRIEST, and ISARIC4C, which further emphasize the greater severity of disease in older adults. The presence of these comorbidities, combined with frailty and reduced physiological reserves, underscores their substantial role in driving worse outcomes, including higher rates of ICU admission and mortality among the elderly.

The clinical utility of these findings is significant, particularly for healthcare settings managing elderly populations vulnerable to severe COVID-19. The demonstration that scores like ISARIC4C, qSOFA, PRIEST, and PAINT can predict severe outcomes means that healthcare providers can implement these tools for the early identification of high-risk patients, potentially directing more aggressive monitoring and therapeutic interventions sooner. For instance, higher scores at admission or the five-day mark could prompt earlier ICU admissions or the initiation of critical care interventions, thus potentially reducing mortality rates. This proactive approach could also facilitate better resource allocation within hospitals, ensuring that medical supplies and personnel are adequately prepared to handle cases likely to require intensive care. Furthermore, these scoring systems can be integrated into triage protocols, helping to streamline patient flow and prioritize treatment for those most likely to benefit from immediate care, thereby enhancing overall hospital efficiency and patient outcomes.

### 4.2. Study Limitations

This study is subject to several limitations that should be considered when interpreting the findings. Firstly, the retrospective design may restrict the ability to establish causal relationships and is susceptible to biases inherent in data collection. The exclusion of certain age groups and individuals with incomplete data may introduce selection bias, affecting the representativeness of the sample. Furthermore, the results derived from a tertiary care setting may not be applicable to primary or secondary healthcare facilities or to areas with varying healthcare resources and protocols for managing COVID-19, thus limiting the generalizability of the conclusions.

## 5. Conclusions

In conclusion, this study reinforces the importance of validated clinical scoring systems in managing COVID-19, particularly among the elderly, who are at increased risk of severe outcomes. The robust predictive power of ISARIC4C, alongside the significant findings of qSOFA, PRIEST, and PAINT, highlight their potential as crucial tools in clinical decision-making processes. Their use not only aids in early detection and management of patients with severe COVID-19 but also optimizes healthcare resources, which is vital during pandemic peaks when hospital capacities can be overwhelmed. Continued research and validation across different populations and settings remain essential to refine these tools and fully realize their potential in global healthcare settings.

## Figures and Tables

**Table 1:** Demographic and clinical characteristics of elderly and control patients.

Variables	>75 Years (*n* = 131)	<65 Years (*n* = 226)	*p*
Age, years (mean ± SD)	78.45 ± 3.12	52.37 ± 9.54	<0.001
Gender, female	79 (60.31%)	109 (48.23%)	0.028
BMI (mean ± SD)	24.78 ± 4.13	27.16 ± 4.87	<0.001
Smoking	19 (14.50%)	62 (27.43%)	0.006
Alcohol use	15 (11.45%)	41 (18.14%)	0.095
COVID-19 vaccinated	70 (53.44%)	120 (53.10%)	0.945
CCI > 2	86 (65.65%)	71 (31.42%)	<0.001
COVID-19 severity			<0.001
Mild	39 (29.77%)	136 (60.18%)	
Moderate	52 (39.69%)	62 (27.43%)	
Severe/critical	40 (30.53%)	28 (12.39%)	
ICU admission	32 (24.43%)	23 (10.18%)	<0.001
Oxygen supplementation	52 (39.69%)	39 (17.26%)	<0.001
Mechanical ventilation	22 (16.79%)	15 (6.64%)	0.003
Mortality	27 (20.61%)	10 (4.42%)	<0.001

BMI—Body Mass Index; SD—Standard Deviation; ICU—intensive care unit; CCI—Charlson Comorbidity Index.

**Table 2:** Baseline clinical scores and physiological parameters at admission.

Variables	>75 Years (*n* = 131)	<65 Years (*n* = 226)	*p*
Oxygen saturation (%)	91.23 ± 3.57	94.58 ± 2.43	<0.001
Respiratory rate (breaths/min)	22.84 ± 3.98	19.37 ± 3.27	<0.001
Heart rate (beats/min)	90.58 ± 11.97	82.22 ± 10.87	<0.001
Temperature (°C)	38.06 ± 0.82	37.77 ± 0.68	0.002
Systolic blood pressure (mmHg)	127.64 ± 17.95	124.97 ± 15.38	0.096
Glasgow Coma Scale	13.84 ± 1.71	14.94 ± 0.38	<0.001
BUN (mg/dL)	32.47 ± 10.69	19.87 ± 8.98	<0.001
Creatinine (mg/dL)	1.46 ± 0.62	0.98 ± 0.44	<0.001
PaO_2_/FiO_2_ ratio	276.52 ± 61.29	318.85 ± 54.16	<0.001
Platelet count (×10^9^/L)	210.46 ± 58.76	244.79 ± 65.32	<0.001
White blood cell count (×10^9^/L)	9.73 ± 3.28	7.58 ± 2.84	<0.001
Clinical scores			
qSOFA	2.18 ± 0.73	1.12 ± 0.61	<0.001
PRIEST	6.52 ± 1.94	3.98 ± 1.47	<0.001
PAINT	7.02 ± 2.08	4.46 ± 1.78	<0.001
ISARIC4C	11.48 ± 3.05	7.02 ± 2.48	<0.001

SD—Standard Deviation; BUN—blood urea nitrogen; PaO_2_/FiO_2_—Arterial Oxygen Partial Pressure to Fractional Inspired Oxygen Ratio; qSOFA—Quick Sequential Organ Failure Assessment; PRIEST—Pandemic Respiratory Infection Emergency Severity Tool; PAINT—Pneumonia Assessment Index for the New Therapy; ISARIC4C—International Severe Acute Respiratory and Emerging Infection Consortium 4C Mortality Score.

**Table 3:** Clinical scores and physiological parameters at five days post-symptom onset.

Variables	>75 Years (*n* = 131)	<65 Years (*n* = 226)	*p*
Oxygen saturation (%)	89.26 ± 4.41	93.12 ± 3.14	<0.001
Respiratory rate (breaths/min)	24.18 ± 4.80	20.14 ± 3.92	<0.001
Heart rate (beats/min)	93.78 ± 13.41	84.09 ± 11.56	<0.001
Temperature (°C)	37.88 ± 0.93	37.41 ± 0.78	<0.001
Systolic blood pressure (mmHg)	125.39 ± 17.84	123.06 ± 15.98	0.223
Glasgow Coma Scale	13.47 ± 2.14	14.89 ± 0.67	<0.001
BUN (mg/dL)	35.23 ± 11.46	21.54 ± 9.32	<0.001
Creatinine (mg/dL)	1.62 ± 0.74	1.02 ± 0.49	<0.001
PaO_2_/FiO_2_ ratio	259.85 ± 68.13	312.46 ± 57.29	<0.001
Platelet count (×10^9^/L)	198.47 ± 61.32	239.52 ± 67.81	<0.001
White blood cell count (×10^9^/L)	11.26 ± 3.71	8.16 ± 3.04	<0.001
Clinical scores			
qSOFA	2.46 ± 0.82	1.22 ± 0.65	<0.001
PRIEST	7.02 ± 2.13	4.46 ± 1.58	<0.001
PAINT	7.48 ± 2.31	4.97 ± 1.89	<0.001
ISARIC4C	12.56 ± 3.22	7.48 ± 2.74	<0.001

SD—Standard Deviation; BUN—blood urea nitrogen; PaO_2_/FiO_2_—Arterial Oxygen Partial Pressure to Fractional Inspired Oxygen Ratio; qSOFA—Quick Sequential Organ Failure Assessment; PRIEST—Pandemic Respiratory Infection Emergency Severity Tool; PAINT—Pneumonia Assessment Index for the New Therapy; ISARIC4C—International Severe Acute Respiratory and Emerging Infection Consortium 4C Mortality Score.

**Table 4:** Stratified analysis of severe COVID-19 outcomes by vaccination status.

Variables	Elderly Vaccinated (>75 Years)	Elderly Unvaccinated (>75 Years)	Control Vaccinated (<65 Years)	Control Unvaccinated (<65 Years)	*p*
Number of Patients (n)	70	61	120	106	
ICU Admission (%)	14.30%	31.10%	6.20%	12.10%	0.002
Oxygen Supplementation (%)	38.60%	59.00%	28.60%	43.40%	0.015
Mechanical Ventilation (%)	9.80%	21.30%	4.20%	10.20%	0.01
Mortality (%)	13.20%	25.40%	3.30%	8.70%	0.003
Mean qSOFA Score ± SD	2.1 ± 0.7	2.6 ± 0.8	1.1 ± 0.6	1.4 ± 0.7	0.043
Mean PRIEST Score ± SD	6.1 ± 1.8	7.5 ± 2.1	3.8 ± 1.4	5.3 ± 1.7	0.021
Mean PAINT Score ± SD	6.3 ± 2.0	8.3 ± 2.2	4.3 ± 1.7	5.9 ± 2.0	0.019
Mean ISARIC4C Score ± SD	11.6 ± 3.1	13.4 ± 3.3	7.2 ± 2.4	9.3 ± 2.7	0.026

SD—Standard Deviation; qSOFA—Quick Sequential Organ Failure Assessment; PRIEST—Pandemic Respiratory Infection Emergency Severity Tool; PAINT—Pneumonia Assessment Index for the New Therapy; ISARIC4C—International Severe Acute Respiratory and Emerging Infection Consortium 4C Mortality Score.

**Table 5:** Optimal cutoff values for predicting severe COVID-19 in elderly patients.

Clinical Score	Time	Optimal Cutoff	Sensitivity	Specificity	AUC	*p*
qSOFA	At admission	2.1	82.14	78.95	0.801	<0.001
PRIEST	At admission	6.3	60.95	77.78	0.730	<0.001
PAINT	At admission	6.5	83.33	79.31	0.812	<0.001
ISARIC4C	At admission	11.8	75.71	81.25	0.772	<0.001
qSOFA	At 5 days	2.5	85.71	70.06	0.733	<0.001
PRIEST	At 5 days	6.2	83.33	80.43	0.823	<0.001
PAINT	At 5 days	7.7	76.11	62.14	0.644	<0.001
ISARIC4C	At 5 days	11.2	88.89	64.62	0.765	<0.001

qSOFA—Quick Sequential Organ Failure Assessment; PRIEST—Pandemic Respiratory Infection Emergency Severity Tool; PAINT—Pneumonia Assessment Index for the New Therapy; ISARIC4C—International Severe Acute Respiratory and Emerging Infection Consortium 4C Mortality Score.

**Table 6:** Regression analysis for severe COVID-19 development in elderly patients.

Score	Time Point	Hazard Ratio	95% CI	*p*
qSOFA > 2.1	At admission	2.52	1.73–3.67	<0.001
PRIEST > 6.3	At admission	2.19	1.50–3.19	<0.001
PAINT > 6.5	At admission	2.85	1.95–4.17	<0.001
ISARIC4C > 11.8	At admission	3.02	2.07–4.41	<0.001
qSOFA > 2.5	At 5 days	2.89	2.02–4.14	<0.001
PRIEST > 6.2	At 5 days	2.38	1.64–3.46	<0.001
PAINT > 7.7	At 5 days	3.12	2.11–4.62	<0.001
ISARIC4C > 11.2	At 5 days	3.28	2.19–4.92	<0.001

qSOFA—Quick Sequential Organ Failure Assessment; PRIEST—Pandemic Respiratory Infection Emergency Severity Tool; PAINT—Pneumonia Assessment Index for the New Therapy; ISARIC4C—International Severe Acute Respiratory and Emerging Infection Consortium 4C Mortality Score.

**Table 7:** Regression analysis for mortality in elderly patients over 75 years after COVID-19.

Score	Time Point	Hazard Ratio	95% CI	*p*
qSOFA > 2.1	At admission	2.47	1.54–3.73	<0.001
PRIEST > 6.3	At admission	2.11	1.41–3.12	<0.001
PAINT > 6.5	At admission	2.60	1.73–3.90	<0.001
ISARIC4C > 11.8	At admission	2.82	1.85–5.24	<0.001
qSOFA > 2.5	At 5 days	2.73	1.83–4.06	<0.001
PRIEST > 6.2	At 5 days	2.36	1.56–3.47	<0.001
PAINT > 7.7	At 5 days	3.34	2.00–4.50	<0.001
ISARIC4C > 11.2	At 5 days	3.32	2.13–5.18	<0.001

qSOFA—Quick Sequential Organ Failure Assessment; PRIEST—Pandemic Respiratory Infection Emergency Severity Tool; PAINT—Pneumonia Assessment Index for the New Therapy; ISARIC4C—International Severe Acute Respiratory and Emerging Infection Consortium 4C Mortality Score.

## Data Availability

The data presented in this study are available upon request from the corresponding author.
